# Open Defecation and Childhood Stunting in India: An Ecological Analysis of New Data from 112 Districts

**DOI:** 10.1371/journal.pone.0073784

**Published:** 2013-09-16

**Authors:** Dean Spears, Arabinda Ghosh, Oliver Cumming

**Affiliations:** 1 Delhi School of Economics, Centre for Development Economics, New Delhi, Delhi, India; 2 Indian Administrative Service, Nadia, West Bengal, India; 3 London School of Hygiene and Tropical Medicine, Environmental Health Group, Department of Disease Control, Faculty of Infectious and Tropical Disease, London, United Kingdom; California Department of Public Health, United States of America

## Abstract

Poor sanitation remains a major public health concern linked to several important health outcomes; emerging evidence indicates a link to childhood stunting. In India over half of the population defecates in the open; the prevalence of stunting remains very high. Recently published data on levels of stunting in 112 districts of India provide an opportunity to explore the relationship between levels of open defecation and stunting within this population. We conducted an ecological regression analysis to assess the association between the prevalence of open defecation and stunting after adjustment for potential confounding factors. Data from the 2011 HUNGaMA survey was used for the outcome of interest, stunting; data from the 2011 Indian Census for the same districts was used for the exposure of interest, open defecation. After adjustment for various potential confounding factors – including socio-economic status, maternal education and calorie availability – a 10 percent increase in open defecation was associated with a 0.7 percentage point increase in both stunting and severe stunting. Differences in open defecation can statistically account for 35 to 55 percent of the average difference in stunting between districts identified as low-performing and high-performing in the HUNGaMA data. In addition, using a Monte Carlo simulation, we explored the effect on statistical power of the common practice of dichotomizing continuous height data into binary stunting indicators. Our simulation showed that dichotomization of height sacrifices statistical power, suggesting that our estimate of the association between open defecation and stunting may be a lower bound. Whilst our analysis is ecological and therefore vulnerable to residual confounding, these findings use the most recently collected large-scale data from India to add to a growing body of suggestive evidence for an effect of poor sanitation on human growth. New intervention studies, currently underway, may shed more light on this important issue.

## Introduction

Sanitation remains a major public health concern with an estimated 40% of the global population lacking access to safe sanitation and 15% still defecating in the open [1]. The failure to effectively contain and manage human excreta is associated with a wide range of health problems and a large disease burden [2,3]. Recent systematic reviews have found that sanitation interventions can be effective in reducing a range of important health outcomes, including diarrhoeal diseases [4,5] and soil-transmitted helminth infections [6]. A number of studies have also suggested that poor sanitation may be associated with adverse nutritional outcomes via different pathways, including diarrhoea [7] and gastro-intestinal disorders such as tropical sprue [8] or tropical enteropathy [9]. In particular, Lin et al [10] have recently shown that children in rural Bangladesh that children who are exposed to worse sanitation are more likely to show indicators of enteropathy and are notably shorter, on average.

As far as the authors are aware, the epidemiological literature offers only three experimental studies to assess the effect of interrupting faecal-oral transmission on stunting [11]. All three of these studies [12,13,14] were cluster-randomised controlled trials to assess the effect of interventions to improve the microbiological quality of drinking water through solar disinfection [12] and the findings have been questioned with regard to plausibility [15] and measurement bias [16]. No epidemiological experimental studies, however, were found for the effect of sanitation or open defecation on childhood stunting.

In the field of economics, however, there has been increasing interest in the relationship between sanitation and human capital in recent years. A series of papers have argued that there exists a causal relationship between open defecation and stunting. In a recent analysis of data from 140 Demographic and Health Survey (DHS) from 65 countries, open defecation explains 54% of the variation in average child height among poor and middle-income countries, and 65% when the population density of open defecation is considered, because poor sanitation is a larger threat when children live nearer to it [17]. A second paper, released by the World Bank Water and Sanitation Program, presents the findings from a cluster randomized controlled trial of a community-level government sanitation program in a district of Maharashtra, India and reports a protective effect on child height adjusted for age [18].

India poses a setting of particular interest in regard to the relationship between open defecation and stunting. Despite significant economic growth in recent years and significant progress on a number of critical human development indicators, such as child mortality [19], over half of the population continues to defecate in the open and stunting persists at very high levels in many parts of India [20]. Various authors have seen in the unusually high levels of these two factors a possible explanation for the so-called “Asian Enigma” of persistently low birth weight and subsequent growth among Indian children despite economic and dietary improvements [21].

This study takes advantage of the first large dataset on the prevalence of childhood stunting in India to be published since India’s 2005 DHS, in order to add to the growing literature on this topic and to test the hypothesis that open defecation is associated with child stunting. This ecological analysis is limited by the available data but nonetheless provides an important opportunity to consider a question of public health significance with updated statistics.

As a secondary analysis, we explore the effect on statistical power of dichotomization of continuous height data into a binary indicator of “stunting.” Although it is well-known theoretically that dichotomization sacrifices power (e.g. [22, 23]), the practice remains quite common in the anthropometry and nutrition literature studying child height, including in the source of the HUNGaMA data primarily used in our analysis.

## Methods

This paper considers the association between the prevalence of open defecation and the district-level prevalence of stunting in 112 districts of India. Published data for district-level stunting is matched with published data on sanitation and other variables to assess whether there is population level correlation after adjustment for potential confounding factors. It was not possible to undertake individual level analysis as individual data had been anonymised and reported values had been averaged by district. All statistical analysis was performed using Stata 12.1 software. Ethical approval was not sought for this secondary analysis of publicly available aggregate data.

### Description of data

Four publicly available datasets were used for this analysis. For the outcome of interest, or dependent variable, prevalence of stunting among children, the HUNGaMA Survey Report [24] was used. This is the most recent large-scale data set that includes data on Indian children’s height. The HUNGaMA survey was conducted by the Naandi Foundation between October 2010 and February 2011, and measured 109,093 children under 5 years old (the same age range as the NFHS-3) in 73,670 households.

There are three important limitations to this data: (1) only district-level averages are provided; (2) only dichotomized stunting rates are reported, and not sample mean child heights or heights-for-age, which mechanically limits the explanatory power of our results; (3) the survey was a non-representative sample of 112 out of 640 Indian districts, selected on the basis of a high prevalence of stunting with an additional few ‘top’ (ie. low stunting prevalence) districts for comparison. As a result of these limitations, our analysis is ecological in nature and limited to the sample of 112 districts reported in the HUNGaMA report. The effect of dichotomization is explored through secondary analysis described below. The next most recent child height data are from the third round of the National Family and Health Survey (NFHS-3, India’s version of the Demographic and Health Survey) and the India Human Development Survey, both collected in 2005.

For the exposure of interest, or independent variable, prevalence of open defecation in the Indian Census Report for 2011 was used. Infant mortality rates are taken from the Annual Health Survey 2010-2011; consumption and calorie data are computed from the 2005 National Sample Survey.

### Statistical analysis

In order to conduct multiple regression analysis of the factors that explain child stunting, we match HUNGaMA stunting data to data from the three other public sources described above. First, the 2011 Indian Census, in principle, surveyed every household in India. District-level census summary reports have been published that report a range of statistics; we use the fraction of households who practice open defecation (ie. those not using any form of sanitation), overall and female literacy rates, and the fraction of households that live in an urban area.

Second, the 2010-11 Annual Health Survey, conducted by the Indian census organization, reports district-level infant mortality rates (IMR), which will be used as an alternative measure of the early-life disease environment, to verify the hypothesized mechanism of the effect of open defecation.

Third, the National Sample Survey (NSS) collects detailed expenditure and consumption data from Indian households. We use this to compute district level average monthly per capita expenditure, a key measure of wealth and socioeconomic status. In addition, NSS data and the calorie conversion factors of Gopalan, et al. [25], is used to compute average per capita daily calorie consumption and average per capita daily cereal calorie consumption, as broad measures of general nutritional status.

For 112 observations, each corresponding to a district, we regress stunting prevalence on the natural log of open defecation, on the infant mortality rate, and on a vector of controls. Thus, the dependent variable is the stunting prevalence as a district-level percentage; we replicate our results using both stunting (percent with height-for-age below -2) as the dependent variable and severe stunting (percent with height-for-age below -3) as the dependent variable. The primary independent, or exposure, variable is the prevalence of households defecating in the open as a district-level percentage of households. In all regressions we estimate heteroscedasticity-robust standard errors and districts are weighted by population size.

To demonstrate robustness, we build our regression results in stages, showing the consequence of adding controls. First, we control for the fraction urban as a quadratic polynomial; urban households are less likely to defecate in the open. Next, we add the economic controls from the NSS that are of policy importance and are likely *a priori* to confound the association of interest: expenditure, calorie consumption, and household size. As per economics convention, the log of expenditure is used. Finally, literacy and female literacy were added as potential confounding factors.

The last step in building the regression model was to add IMR. The addition of IMR to the regression model is not intended as a further control, but instead as a test of the hypothesized mechanism linking open defecation to child height: the early-life disease environment. If open defecation indeed causes stunting due to fecal contamination of the environment – a possibility it is beyond the scope of this paper to demonstrate – then open defecation and IMR should be colinear. Moreover, adding a control for IMR should reduce the coefficient on open defecation, because it will absorb some of the true effect of the latent, unobserved disease environment.

**Figure 1:**
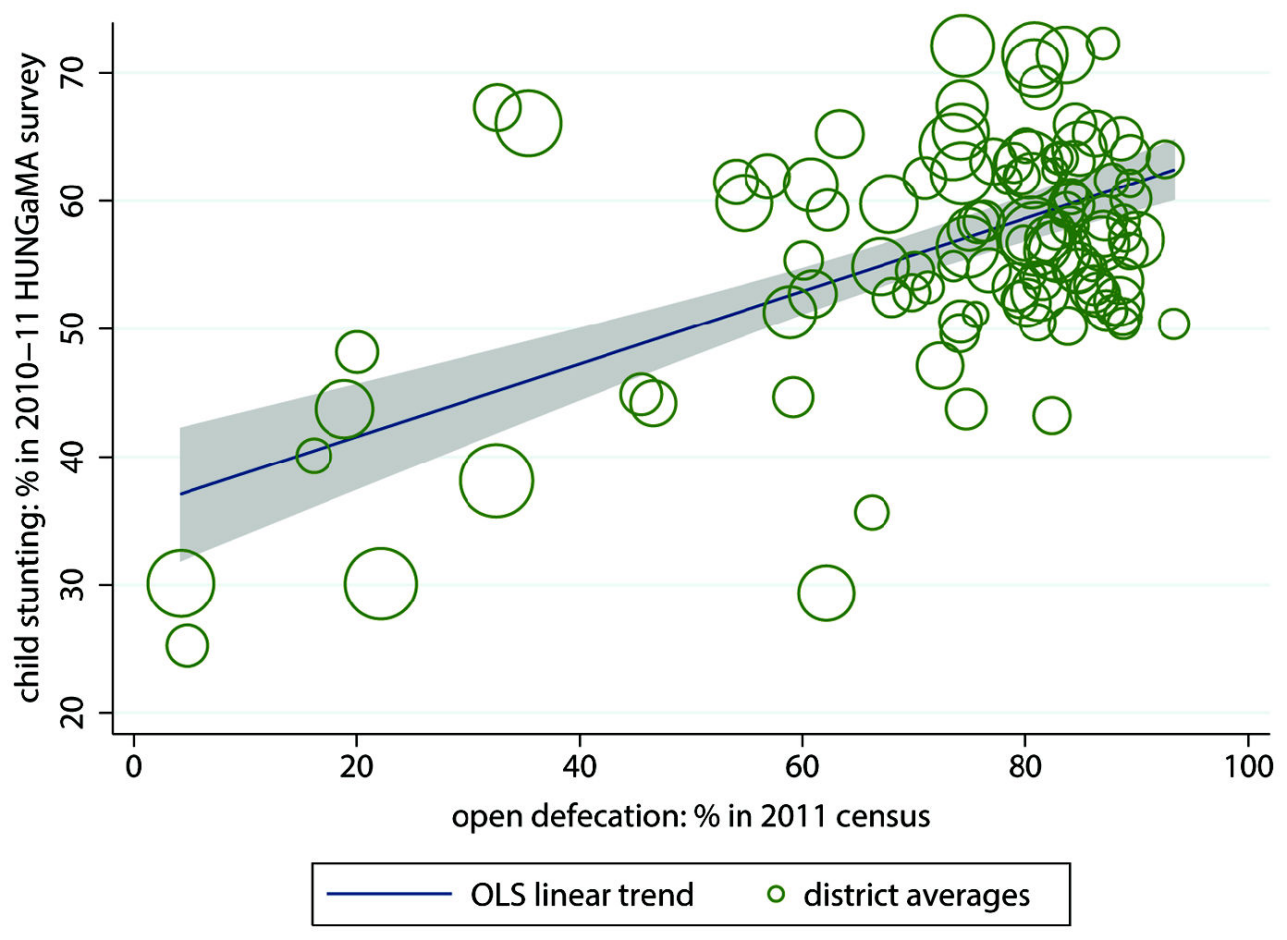
Open defecation predicts stunting, bivariate linear regression. Note: *n* = 112 Indian districts; R^2^ = 34.8%. The size of the circles is proportionate to the population of the districts they represent. The grey shaded area is the 95% confidence set for the regression line.

Finally, open defecation is transformed by natural log, as the recommendation of estimating a Box-Cox transformation. In this small and non-random sample, model fit is important. There is no *a priori* reason to believe that every percentage point reduction in open defecation must reduce stunting rates by the same amount. Moreover, open defecation has a skewness of -2.0 (compared with -1.0 for percent stunted and -0.3 for percent severely stunted). Therefore, we first estimate a Box-Cox transformation of open defecation to select the appropriate shape of the function mapping sanitation to child stunting. This procedure fits a model by transforming open defecation as (*open defecation* ^λ^ -1)/λ, which becomes ln(*open defecation*) if λ = 0, and estimates the parameter λ in addition to the regression coefficients. A maximum likelihood estimation of a Box-Cox transformation of open defecation as an independent variable explaining the fraction of children stunting finds that a natural log transformation is appropriate. In particular, the maximum likelihood estimate of λ is -0.045 (95% confidence interval -0.60 to 0.51). Likelihood ratio tests reject that λ equals -1(*p* = 0.003) or 1 (*p* = 0.005) but do not reject that λ equals 0. Therefore, open defecation is transformed as ln(*open defecation*), corresponding with λ = 0. Note that while this transformation improves model fit, Figure 1 below shows that open defecation is also associated with child stunting without the transformation.

Finally we separately explored the effect of dichotomization of stunting on statistical power. We used the 41,306 observations of children under 5 years old measured in the NFHS-3 to conduct a Monte Carlo simulation. Random subsets were drawn of the sample of children with measured height; within each subset we estimated the association between local open defecation rates and both average child height-for-age and stunting rates. Thus, the following procedure was repeated 1,000 times:

1 a simple random sample of 20,000 children was drawn;
2 for this sample, a primary sampling unit (PSU) average (ie. collapsed mean) was computed for fraction of households practicing open defecation, average height-for-age, and fraction of children stunted and severely stunted;
3 for the collapsed sub-sample of PSU-level means, three regressions were estimated of mean height-for-age, fraction stunted, and fraction severely stunted, each as dependent variables, on the PSU fraction of households practicing open defecation, as the independent variable;
4 the regression estimates, *t*-statistics, and *R* ^2^, were recorded and then the procedure repeated with a new random subsample.

This analysis permits a comparison of the *R* ^2^ and *t*-statistics across dichotomized and non-dichotomized specifications using the same subsample of children from the NFHS-3.

## Results

Table 1 presents sample means for the variables used in this paper. In general, children in these districts are unhealthy, and households are poor. Over half of the children are stunted, and almost a third of children are severely stunted (3SD). The early-life disease environment is poor: over 70 percent of households defecate in the open and 71 out of every 1,000 babies born alive die before they are 1 year old. Two-thirds of all adults, and slightly more than half of females, are reported as literate in the census.

**Table 1:** Descriptive statistics.

variable	mean	min	max	data source
stunting (height < -2 s.d.), percent	55.9	25.2	72.3	HUNGaMA survey, 2010-11
severe stunting (height < -3 s.d.), percent	31.6	10.9	50.3	HUNGaMA survey, 2010-11
open defecation, percent	70.5	4.2	93.3	Indian census, 2011
infant mortality rate	71.3	35.7	103.0	Annual Health Survey, 2010-11
urban residence, percent of households	18.8	3.4	75.8	Indian census, 2011
literacy rate, overall	66.4	44.5	96.9	Indian census, 2011
literacy rate, female	55.8	34.2	96.3	Indian census, 2011
monthly per capita expenditure, Rupees	563	284	1,573	National Sample Survey, 2005
calories per capita, per day	2,056	1,573	2,612	National Sample Survey, 2005
cereal calories per capita, per day	1,411	1,006	1,968	National Sample Survey, 2005
household size	6.3	4.0	8.6	National Sample Survey, 2005

**Figure 2:**
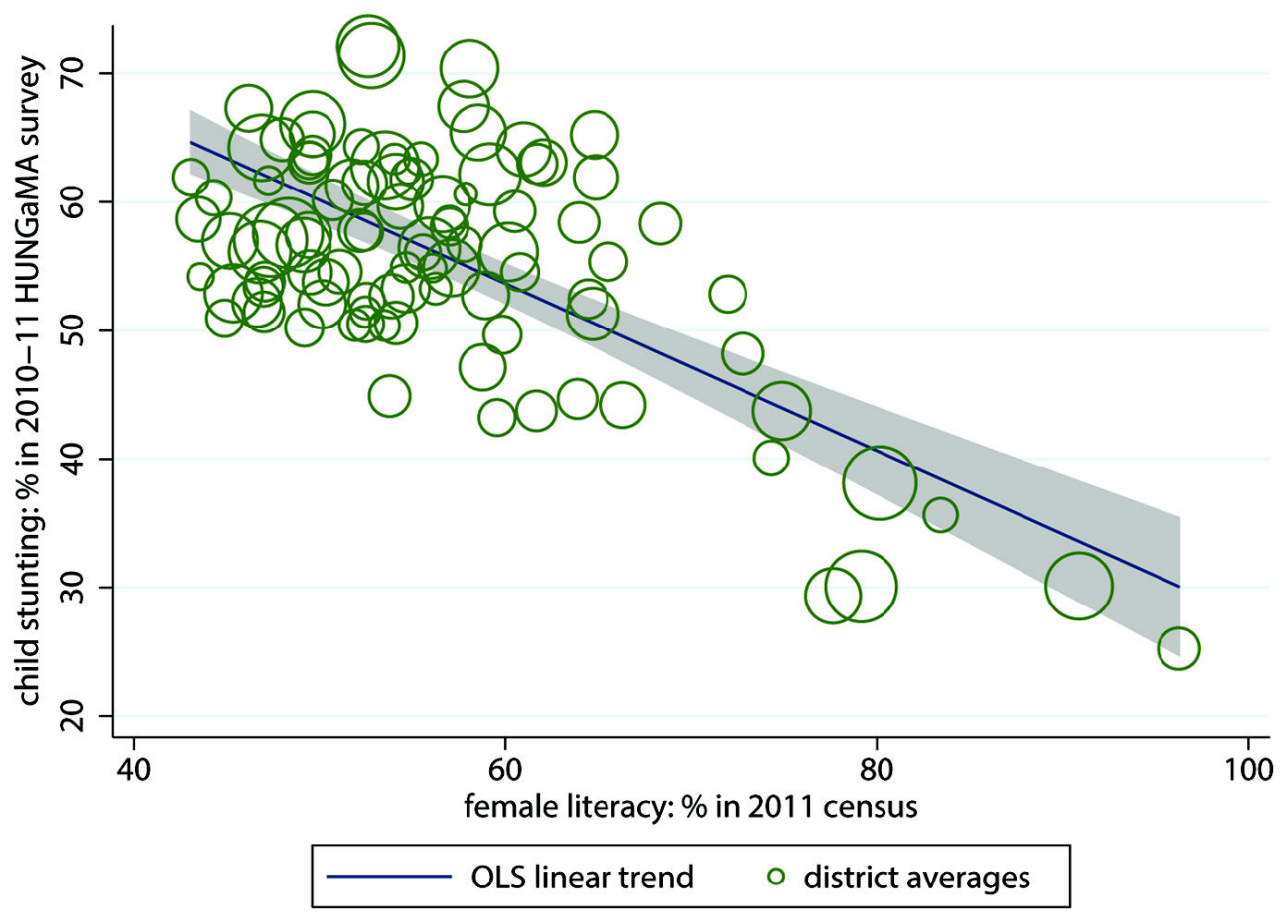
Female literacy predicts stunting, bivariate linear regression. Note: *n* = 112 Indian districts; R^2^ = 48.5%. The size of the circles is proportionate to the population of the districts they represent. The grey shaded area is the 95% confidence set for the regression line.

**Figure 3:**
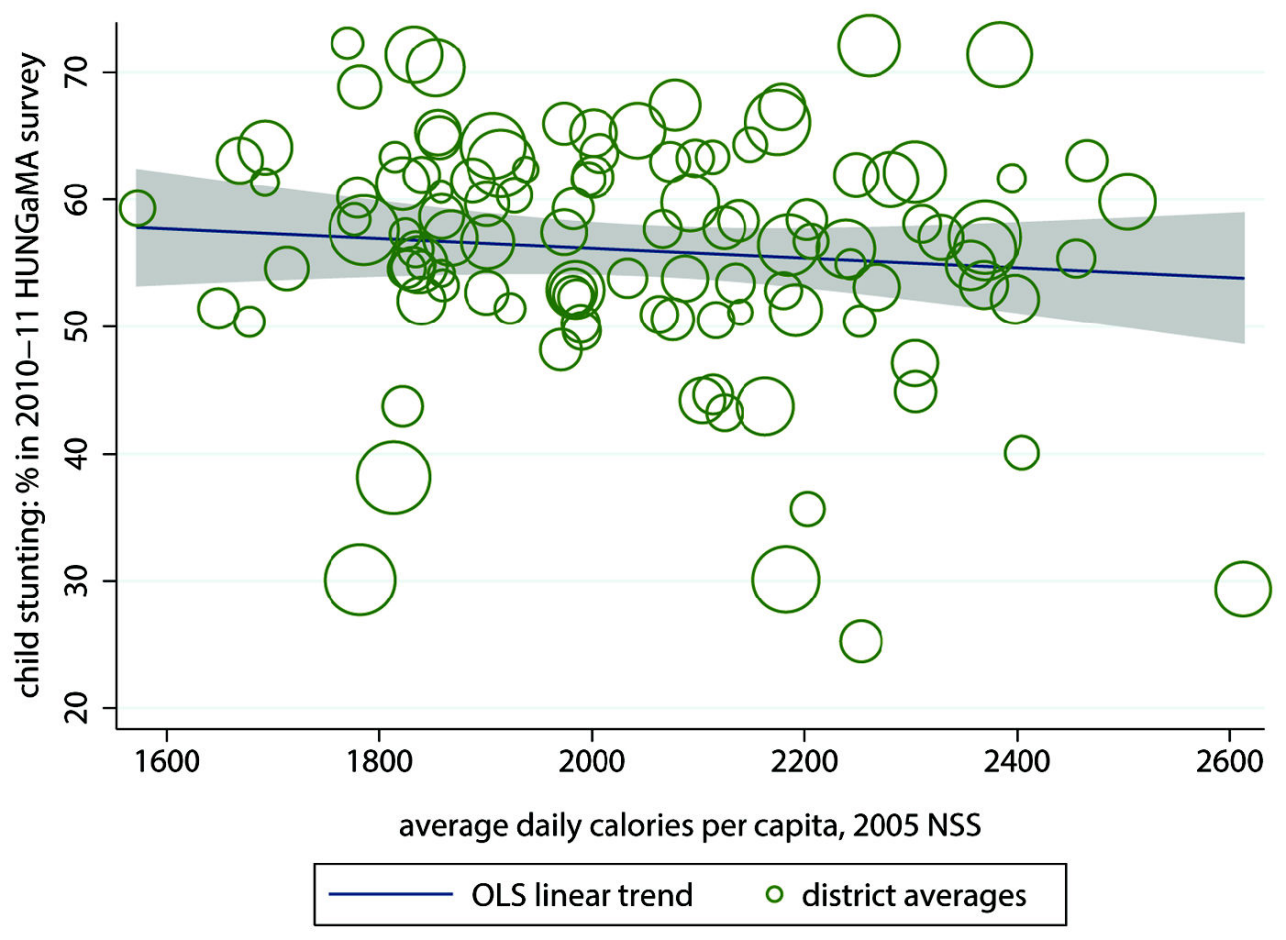
District average calorie consumption does not predict stunting, bivariate linear regression. Note: *n* = 112 Indian districts; R^2^ = 0.7%. NSS = National Sample Survey. The size of the circles is proportionate to the population of the districts they represent. The grey shaded area is the 95% confidence set for the regression line.

Among these districts, which variables are correlated with child stunting? Figure 1 shows that districts with more open defecation also have more stunting; the *R* ^2^ is 34.5%. Female literacy, often used as an indicator for women’s social status more generally, also predicts child height, as shown in Figure 2. Districts with higher rates of female literacy have less stunting, on average, with an *R* ^2^ of 48.5%. This is consistent with the recent finding of Coffey, et al. [30] that within rural Indian joint households, children of lower-ranking mothers are shorter, on average. In contrast, average calorie consumption does not predict district-level stunting rates, as Figure 3 shows. The *R* ^2^ is near 0, and there is no visible trend.

Does the relationship between sanitation and stunting hold in a multiple regression with controls? In particular, given that open defecation and female literacy are both statistically significant correlates of child stunting, this section explores how they and other control variables combine in a single regression.

Tables 2 and 3 report regression results and should be read together as replications of one another. Column 1 shows that districts with more open defecation have more stunted children. The fall in the coefficient moving to column 2 suggests that some, but not all, of this correlation reflected omitted heterogeneity in urbanization across districts. However, the coefficient changes very little (in some cases becoming larger) as economic controls and literacy controls are added. In particular, neither calorie consumption nor female literacy is statistically significant in the fully controlled regressions (column 4). A ten percent increase in open defecation is associated with about a 0.7 percentage point increase in both stunting and severe stunting.

**Table 2:** Explaining district-level variation in child stunting (< -2 standard deviations), OLS.

	(1)	(2)	(3)	(4)	(5)
	percent of children stunted, OLS				
ln(open defecation)	11.02**	7.969**	8.628**	7.082*	5.228†
	(1.550)	(1.597)	(2.472)	(2.803)	(2.729)
IMR					0.145*
					(0.0589)
percent urban		0.216	0.128	0.246	0.139
		(0.218)	(0.216)	(0.173)	(0.181)
percent urban²		-0.00504†	-0.00480†	-0.00512*	-0.00336
		(0.00290)	(0.00274)	(0.00229)	(0.00242)
ln(mpc expenditure)			8.765	8.103	8.718
			(7.816)	(6.986)	(6.664)
calories per capita			-0.0119	-0.00466	-0.00668
			(0.0102)	(0.00907)	(0.00874)
cereal calories per			0.00235	-0.00476	-0.000828
capita			(0.00816)	(0.00782)	(0.00776)
household size			1.470	1.595†	1.538†
			(1.077)	(0.852)	(0.815)
literacy, overall				-0.810†	-0.671
				(0.450)	(0.444)
literacy, female				0.335	0.243
				(0.461)	(0.447)
constant	10.10	22.09**	-22.28	15.80	4.993
	(6.836)	(7.647)	(46.13)	(43.93)	(41.78)
*n* (districts)	112	112	112	112	110
*R* ^2^	0.389	0.453	0.484	0.586	0.617

Note: Robust standard errors in parentheses. Two-sided *p*-values: † *p* < 0.10, *p < 0.05, **p < 0.01. MPC expenditure means monthly per capita expenditure. IMR means infant mortality rate.

**Table 3:** Explaining district-level variation in severe child stunting (< -3 standard deviations), OLS.

	(1)	(2)	(3)	(4)	(5)
	percent of children severely stunted, OLS				
ln(open defecation)	8.599**	5.293**	6.699**	6.646**	4.318†
	(1.227)	(1.105)	(2.186)	(2.476)	(2.277)
IMR					0.174**
					(0.0478)
percent urban		0.136	0.0366	0.138	0.0123
		(0.166)	(0.168)	(0.133)	(0.138)
percent urban²		-0.00409†	-0.00388†	-0.00422*	-0.00214
		(0.00216)	(0.00207)	(0.00173)	(0.00175)
ln(mpc expenditure)			11.18	11.07†	11.58*
			(7.053)	(6.215)	(5.706)
calories per capita			-0.0120	-0.00544	-0.00798
			(0.00872)	(0.00801)	(0.00752)
cereal calories			0.00449	-0.00247	0.00257
per capita			(0.00748)	(0.00711)	(0.00670)
household size			1.188	1.539*	1.416*
			(0.918)	(0.756)	(0.700)
literacy, overall				-1.043*	-0.852*
				(0.416)	(0.389)
literacy, female				0.636	0.506
				(0.409)	(0.382)
constant	-4.126	9.789†	-53.70	-26.47	-37.90
	(5.312)	(5.285)	(43.01)	(39.14)	(35.88)
*n* (districts)	112	112	112	112	110
*R* ^2^	0.312	0.391	0.424	0.533	0.592

Note: Robust standard errors in parentheses. Two-sided *p*-values: † *p* < 0.10, *p < 0.05, **p < 0.01. MPC expenditure means monthly per capita expenditure. IMR means infant mortality rate.

Does the association between open defecation and child height indeed reflect the early-life disease environment? If so, then the coefficient should be reduced by adding another measure of the disease environment. Column 5 adds IMR, which has a correlation of 0.47 with the natural log of open defecation in this sample. Note that this is an intentionally incorrectly specified model, for the purpose of seeing the effect on the coefficient on open defecation. Indeed the coefficient on open defecation falls (but does not lose statistical significance at the two-sided 0.10 level), suggesting that the association is indeed due to the early-life disease environment.

We find that open defecation and female literacy both predict stunting rates in bivariate regressions, but calorie consumption does not. In multiple regressions, open defecation is the key predictor of district-level stunting.

**Figure 4:**
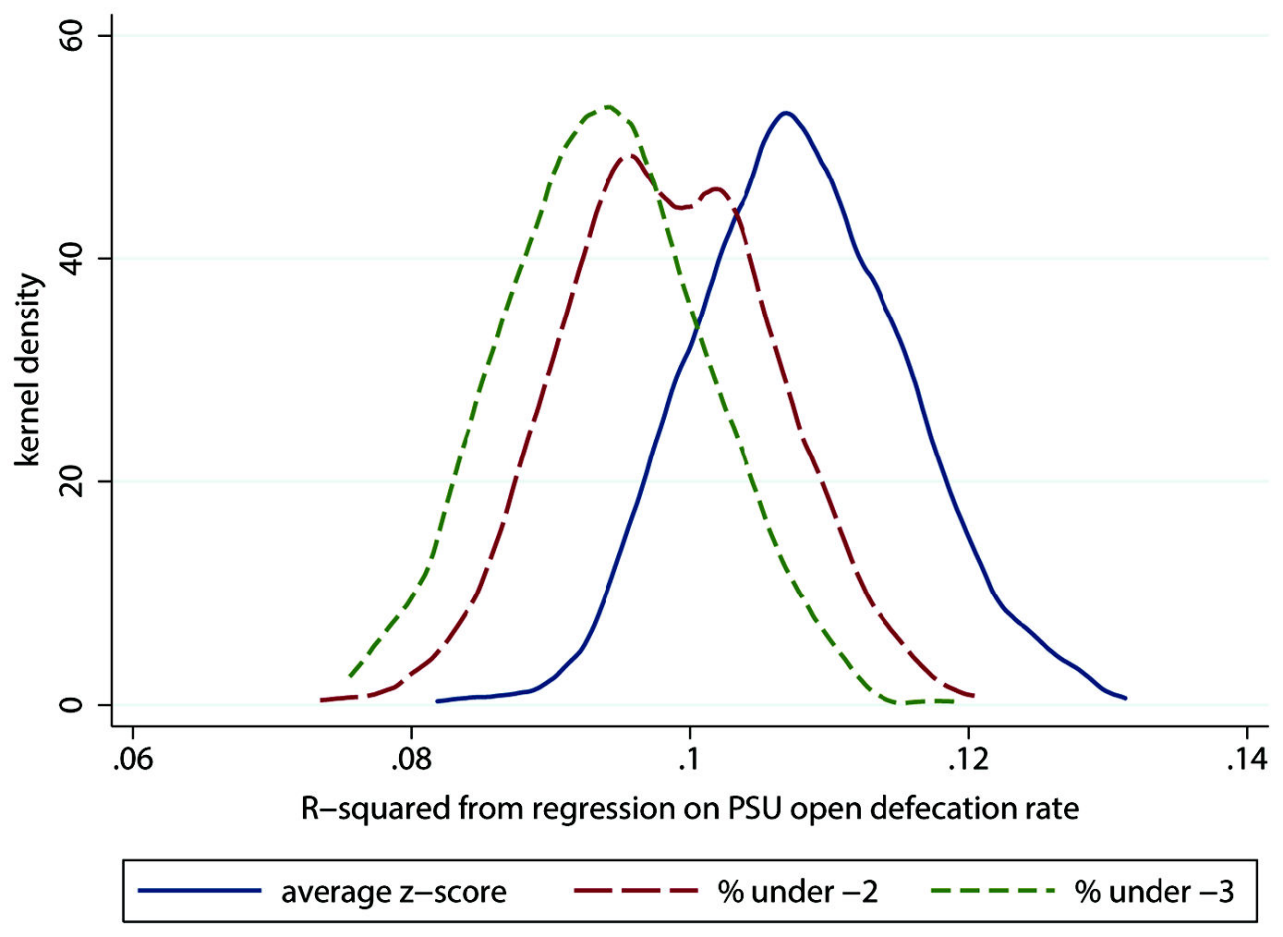
Dichotomization reduces statistical power: *R* ^*2*^, simulations using NFHS-3. Note: Observations are 1,000 Monte Carlo samples of 20,000 children under 5 drawn from India’s 2005 National Family and Health Survey. PSU = survey primary sampling unit (local area). The legend reports regression dependent variables.

**Figure 5:**
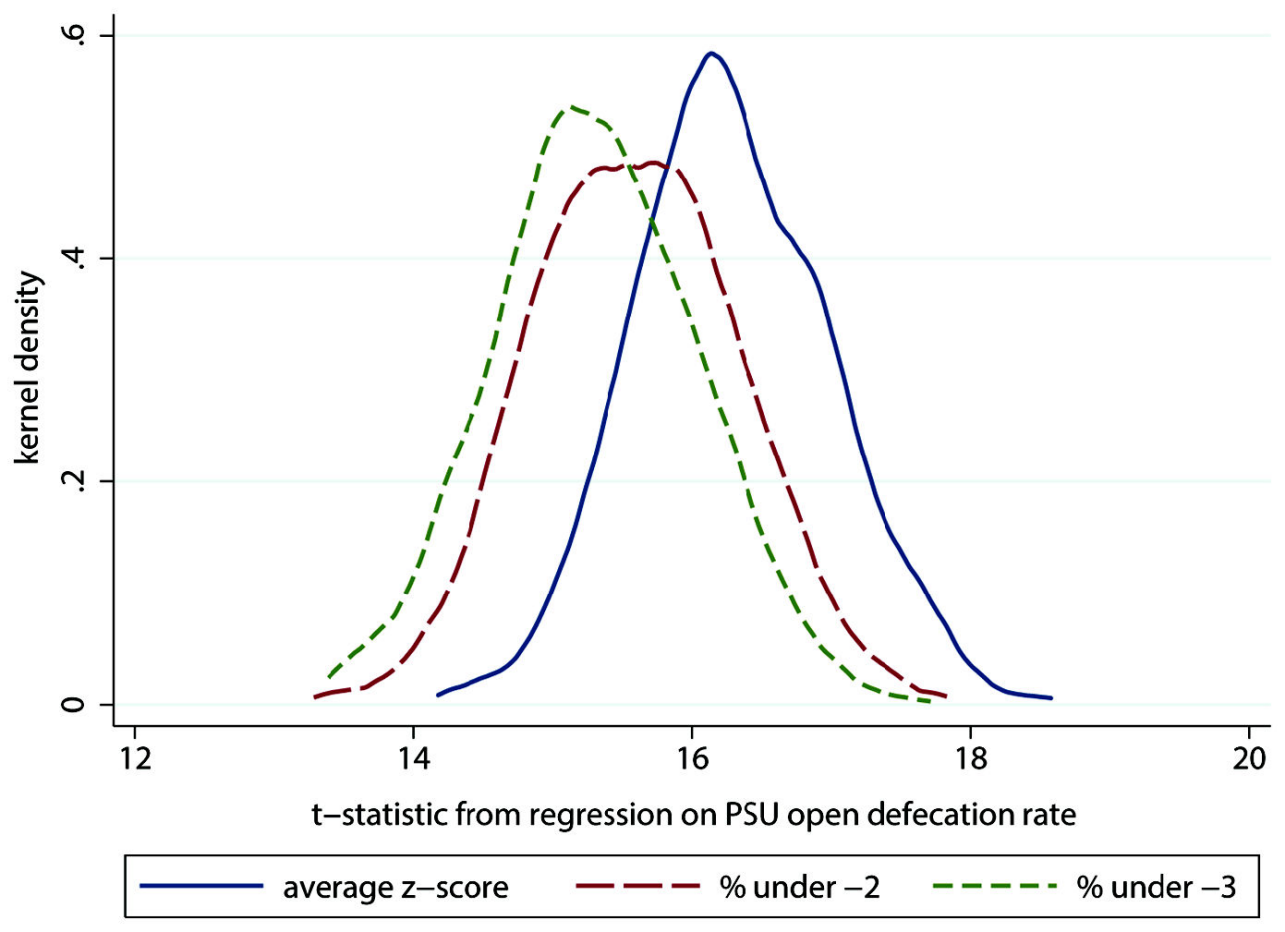
Dichotomization reduces statistical power: *t*-statistics, simulations using NFHS-3. Note: Observations are 1,000 Monte Carlo samples of 20,000 children under 5 drawn from India’s 2005 National Family and Health Survey. PSU = survey primary sampling unit (local area). The legend reports regression dependent variables.

Lastly we considered whether statistical power is lost in our analysis due to the dichotomization of child height data in the HUNGaMA survey. For the 1,000 Monte Carlo simulations, Figure 4 presents the distribution of regression *R* ^2^ s, and Figure 5 plots the distributions of *t*-statistics. In both cases, the distribution when the non-dichotomized mean of height-for-age *z*-scores is used as the dependent variable is to the right, indicating the greatest statistical power. Out of 1,000 randomly drawn samples, the *R* ^2^ is greater with the non-dichotomized dependent variable than with both dichotomized dependent variables in 955 cases, and the *t*-statistic is greatest in the non-dichotomized specification in 881 cases. For child height – as has been documented for other variables – dichotomization sacrifices power. This suggests that our findings with the HUNGaMA data may be a lower bound on the true power of open defecation to explain variation in Indian children’s height.

## Discussion

How quantitatively important is our estimate that a one unit increase in the log of the percent of a district’s population defecating in the open is associated with a 7 percentage point increase in child stunting? To assess the magnitude of this, recall that the HUNGaMA sample of 112 districts was selected to include 100 low performing districts and 12 high performing districts. On average, 59.1 percent of children in the low performing districts are stunted, compared with 35.9 percent of children in the high performing districts, a 23 percentage point difference. Similarly, in the 2011 census, 76.3 percent of households reported defecating in the open in the low performing districts, compared with 33.8 percent – still a large fraction – in the high performing districts. Our regression estimates suggest that this difference in open defecation can account for 35 to 55 percent of this gap in stunting prevalence between high and low performing districts (corresponding to the estimates in columns 4 and 1 of Table 2, respectively) – which, again, may be lower bounds of the true explanatory power of open defecation, due to the dichotimization of height.

This significance of the findings from this analysis is limited by the underlying limitations of the available data. The advantage of this data is that it is the only large-scale data collected on Indian children’s height since 2005. The three major limitations of the data – that only district-level averages for stunting were available; that dichotomized stunting rates are presented, not sample means; and that the sample of districts is not randomly selected – are all discussed above and to a greater extent determine the analytical approach undertaken. Although open defecation robustly predicts heterogeneity in child stunting across Indian districts in our analysis of this data, our findings must be viewed in the context of this being an ecological analysis of a deliberate sample of 112 districts in 2010-11, and not as an estimate of any causal effect of open defecation on child height.

Ecological analysis is often used to generate hypotheses for further investigation using more rigorous methods. In this instance, we instead sought to further assess an existing hypothesis – that open defecation is an important cause of child stunting – in India where this hypothesis may have particular public health importance. Of the over 1 billion people who practice open defecation in the world, over 600 million reside in India [1]; of about 215 million children who are stunted today, 28.5% reside in India [26]. A hypothesis linking these two public health challenges of high levels of open defecation and stunting is therefore of particular relevance to India. The recently released HUNGaMA survey report, when used in conjunction with other publicly available data, provided such an opportunity to assess whether high levels of open defecation were associated with high levels of stunting in India.

This study sheds no light on the mechanism by which an association between open defecation and stunting, if causal, might operate. There are at least three plausible pathways – repeated bouts of diarrhea, intestinal worms (in particular, soil transmitted helminth infections, hookworm, *Trichuris* , and ascaris), and environmental enteropathy – that have been suggested. A multi-country analysis found that far more variation was explained by poor sanitation and water than by repeated bouts of diarrhea [7], suggesting that other pathways may be important, such as enteropathy [10]. It would have been preferable in this analysis to explore this further by controlling for these variables – in particular, prevalence of diarrhoeal diseases and helminth infection – but this was beyond the limits of the available data.

As highlighted by Humphrey [9], a meta-analysis of 38 studies for food programmes in developing countries found that the best results achieved were 0.7 of a standard deviation for height-for-age [27] suggesting that food intake alone does not explain the deficit in growth in developing countries. Unicef, in its nutrition framework, calls for ‘nutrition sensitive’ interventions that will support and enhance ‘nutrition’ or food-based interventions to reduce stunting [28]. The analysis presented here supports the consideration of sanitation as one such ‘nutrition sensitive’ intervention. Further work though is required to both establish a causal effect, especially of any particular intervention scheme, as well as to identify the most effective approaches for targeting and delivering this intervention.

Of particular interest for policy in India is that, in a crude analysis of association, calorie consumption did not predict higher levels of stunting. This is consistent with the observation that calorie consumption has been declining in India, despite high levels of malnutrition measured as stunting [29]. Our results, in the context of this literature, suggest that mere provision of calories *per se* is unlikely to importantly reduce stunting among Indian children [30].

Finally, and as something of a methodological footnote, our assessment of the effect of dichotomisation on statistical power confirms others’ findings that this sacrifices statistical power. Despite clear methodological recommendations in the literature, it remains common in the nutrition policy literature (such as the HUNGaMA report) to focus on dichotomized anthropometric indicators, such as stunting. Our results suggest that average height would be a more informative summary.

## Conclusion

Stunting is a persistent public health challenge in many countries, and in particular in India, and more effective strategies are needed. A failure to reduce stunting limits the development prospects of individuals and exacts a heavy cost on economic productivity, thereby further limiting the development prospects of low income countries. Whilst not conclusive, this analysis adds to a growing body of suggestive evidence for the effect of open defecation or poor sanitation – and the disease environment more generally [31] – on human growth. There is already an urgent and compelling need for progress on sanitation in India, and in many other countries, on the basis of other associated health concerns and its recent recognition as a human right. New and emerging evidence – including the analysis presented here – suggests that the priority afforded to sanitation must now also take account of its potential contribution to the enduring challenge of childhood stunting.
