# Correction: Open Defecation and Childhood Stunting in India: An Ecological Analysis of New Data from 112 Districts

**DOI:** 10.1371/annotation/9ffcb740-f394-41af-bbbc-800c7cc25ea8

**Published:** 2013-09-23

**Authors:** Dean Spears, Arabinda Ghosh, Oliver Cumming

Information regarding the involvement of one of the funding organizations was incorrectly omitted from the Funding Statement. The following sentence should be included as the fourth sentence of the Funding Statement: "However, the views expressed in this paper belong to the authors and do not necessarily reflect DfID’s official policies or the policies of WSSCC or the Gates Foundation, nor do they necessarily reflect the position of the Indian Government." 

